# Nerve Cell Responses to Biocompatible RGO–Chitosan–CMC Nanofiber Composite Membranes

**DOI:** 10.3390/ijms27188013

**Published:** 2026-09-09

**Authors:** Quentin Sins, Giulia Zo, Giuliana Paravizzini, Elena Raluca Sandu, Stefania Raimondo, Wang Meng-Jiy, Federica Fregnan, Yuki Shirosaki

**Affiliations:** 1 Department of Engineering, Graduate School of Engineering, Kyushu Institute of Technology, 1-1 Sensui-cho, Tobata-ku, Kitakyushu-shi 804-8550, Fukuoka, Japan; 2 Department of Clinical and Biological Sciences, and Cavalieri Ottolenghi Neuroscience Institute, University of Turin, Regione Gonzole 10, 10043 Orbassano, Italystefania.raimondo@unito.it (S.R.); federica.fregnan@unito.it (F.F.); 3 Department of Chemical Engineering, National Taiwan University of Science and Technology, No. 43, Sec. 4, Keelung Rd., Taipei 106, Taiwan; mjwang@gapps.ntust.edu.tw; 4 Department of Materials Science, Faculty of Engineering, Kyushu Institute of Technology, 1-1 Sensui-cho, Tobata-ku, Kitakyushu-shi 804-8550, Fukuoka, Japan; 5 Collaborative Research Centre for Green Materials on Environmental Technology, Kyushu Institute of Technology, 1-1 Sensui-cho, Tobata-ku, Kitakyushu-shi 804-8550, Fukuoka, Japan

**Keywords:** nanofiber, chitosan, carboxymethylcellulose, film, electroconductivity, nerve cell, cell proliferation, cell morphology

## Abstract

Peripheral nerve injuries are common traumas that are difficult to overcome. Although nerve grafting can quickly recover nearly all sensitivity and function, graft availability remains an issue. Research has focused on ways to directly rebuild and promote the healing of damaged nerves using surgically implanted scaffolds. Nerve regeneration can be further promoted through strategies aimed at enhancing the regenerative microenvironment, such as improving the electrical conductivity of nerve guidance conduits. In this study, composite films of chitosan (Ch) and carboxymethyl cellulose (CMC) nanofibers were prepared. To improve the films’ conductivity, reduced graphene oxide (RGO) was added. Structural analysis indicated interactions between Ch and CMC and showed that incorporation of RGO did not produce additional detectable crystalline phases. ChCMCRGO films showed slightly higher conductivity and greater surface hydrophilicity than ChCMC membranes and supported greater neurite outgrowth.

## 1. Introduction

Injuries to the peripheral nervous system are common traumas that can be caused by various mechanical, chemical, or thermal insults (e.g., during work-related tasks) and can result in motor and sensory deficits if the patient is not treated quickly [1]. Recent estimates from the Global Burden of Disease 2021 study indicate that nerve injuries accounted for approximately 4.13 million incident cases worldwide in 2021 and approximately 0.44 million years lived with disability (YLDs). These data highlight the substantial and persistent disability burden associated with peripheral nerve injuries [2]. Although some nerve injuries can heal naturally, severed nerves require surgical reconnection to prevent irreversible loss of sensory and motor function. If the injury is minor, such surgery may be sufficient, but it can result in neuroma or be ineffective if not performed perfectly [3]. For more severe injuries, the “gold standard” treatment has traditionally involved replacing the damaged nerve with a fresh one from the same person (autograft) or from a donor (allograft) [4]. Although these methods are effective, they can cause trauma and sensory loss in the donor zone, neuroma at the site of repair, and rejection in the case of an allograft.

Through advances in tissue engineering, novel methods have been developed to overcome the drawbacks of allografts and autografts while achieving comparable results. Nerve conduits have been developed to protect the injured area from external factors in the body without affecting the activity of the cells involved in regeneration. Initially hollow conduits have since been developed with various lumen structures to enhance regeneration by creating a suitable medium for the cells involved. Such media include nanofibers and hydrogels, which mechanically support the cells, and micro- and nanotubes that guide the regenerated axon. A further development has been the addition of chemical cues, such as nerve growth factors, to the conduit or the lumen [5] to mimic their natural release and stimulate regeneration. Other chemical cues can also be used to achieve comparable or more specific activity.

To create the ideal conduit, materials must be biocompatible to avoid rejection by the body and cellular toxicity, biodegradable to avoid subsequent surgical removal, permeable so that oxygen and nutrients can be exchanged between the injury zone and the external medium, and flexible so that the conduit will not interfere with the regenerated axon if some movement occurs [6]. At first, synthetic materials such as silicone gel, polylactide, or polyglycolide were used, but these led to issues such as nerve compression or generation of acidic degradation products [4]. Subsequent research focused on biopolymer conduits, such as collagen, cellulose, alginate, and chitosan (Ch), with the latter being the material most commonly considered for this application. Ch is a natural semicrystalline polysaccharide derived from chitin. As a biocompatible material, Ch is used to create scaffolds, hydrogels, fibers, and films for medical applications. Rinaudo reported its potential use as a surgical suture, artificial skin, or encapsulating material for time-release drugs [7].

For tissue engineering, Ch possesses the required properties: biocompatibility to avoid an immune reaction, surface characteristics such as porosity to enable cells to adhere and proliferate, mechanical strength to avoid failure before the new tissue is established, and ease of manufacture [8]. It can also be easily chemically modified to incorporate an active ingredient or to crosslink it with another material. Its cationic amine groups can also be used to electrostatically bind it to some materials. These properties have led to the widespread biomedical use of Ch. Accordingly, chitosan-based nerve-guiding conduits have demonstrated excellent biocompatibility, favorable interactions with Schwann cells, and the ability to support axonal regeneration in both experimental and clinical studies [9].

For peripheral nerve regeneration, Xu et al. crosslinked Ch to hyaluronic acid to obtain an injectable hydrogel loaded with nerve growth factors for a slowly degrading filling in implantable nerve-guiding conduits [10]. As a conduit filling, this hydrogel led to better in vivo recovery of nerve defects in rats than the hollow conduit alone. Carboxymethyl cellulose (CMC) was used as a complement to Ch in the following study. CMC is an anionic polysaccharide derived from cellulose, the main component of plants, making it an abundant resource. CMC can be produced by substituting hydrogen in some hydroxyl groups (–OH) of cellulose with a carboxymethyl group (–CH_2_COOH), which makes the macromolecule anionic [11]. CMC has been widely used in various domains, either alone or alongside other materials. It has been used in the textile industry as a dye thickener because of its water-absorbing properties and incorporated into textiles owing to its film-forming properties. In wastewater treatment, its non-toxic and adsorbent properties make it a good candidate for collecting dyes and ions. Being a non-toxic, odorless, and non-caloric material, it has been used instead of gums and alginate as a thickener and emulsifier in the food industry. Its film-forming properties and biocompatibility also make it a good material for food packaging. Finally, CMC has been extensively used in biomedical applications because of its facile cell adhesion, low cytotoxicity, excellent biocompatibility, biodegradability, and cell viability. For example, Capanema et al. demonstrated that a hydrogel comprising CMC and polyethylene glycol was mechanically strong, superabsorbent, and cytocompatible, making it potentially useful as a wound healing agent [12].

Despite its favorable properties, very few studies have addressed the use of CMC as a conduit or filling. Its high water uptake likely makes it an unsuitable conduit material; however, in combination with hyaluronic acid (Seprafilm^®^), CMC is able to prevent axon outgrowth and neuroma formation [13]. In this research, nanofibers from both CMC and hyaluronic acid were formed using the method described by Dutta et al. [14]. The use of nanofibers results in a fibrous structure that is more porous and thus promotes cell proliferation. The viscosity, crystallinity, and tensile strength of Ch nanofibers increase during a low number of passes of Ch solution through a high-pressure water jet.

Among the cues proven to enhance nerve regeneration, electrical stimulation has drawn interest. Al-Majed et al. showed that 1 h of 20 Hz electrical stimulation immediately after surgical repair of a severed rat femoral nerve can repair a gap of 25 mm within 3 weeks instead of 8–10 weeks without stimulation [15]. Long-term (1–2 weeks) stimulation was also equally effective. The electrical current was run through a wire looped around the nerve stump, and the anode and cathode were sutured close to the nerve. Electrical stimulation also increased the functional recovery of the injured nerve, with very few axons connecting to the wrong muscle. Such findings show the potential for electrical stimulation to accelerate nerve regeneration. However, this method requires the use of conductive materials and should not involve more surgery. Recently, among conductive materials, the polymer poly(3,4-ethylenedioxythiophene):polystyrene sulfonate (PEDOT:PSS) has gained significant attention [16] thanks to its excellent electrochemical performance, biocompatibility, and mechanical flexibility, demonstrating it to be a promising material for treating peripheral nerve injuries [17]. Traditional conductive materials such as metals have biocompatibility issues. An alternative conductive and biocompatible material that can be easily solubilized and mixed with polymers is reduced graphene oxide (RGO). This material is obtained from graphite, a crystalline form of carbon made of stacked graphene sheets comprising a hexagonal lattice of carbon atoms. Graphene oxide can be produced from conductive graphite through exfoliation and oxidation. However, the sp^2^ bonding orbitals are disrupted in graphene oxide, making it an electrical insulator [18]. Graphene-like conductivity can be obtained by reducing graphene oxide to restore the sp^2^ bonding orbitals, and since the reduction does not completely remove all oxygen groups, the resulting RGO material remains. Soluble RGO has gained interest as a conductive filling for polymer materials. For example, coating a fibroin/poly(L-lactide-*co*-*ε*-caprolactone) nanofiber scaffold with RGO greatly enhanced Schwann cell migration, proliferation, and myelination around the scaffold, and it achieved a conductivity as high as 0.0–0.04 S/m during electrical stimulation that enhanced the differentiation of NC12 cells in vitro [19].

In this study, we prepared ChCMCRGO composite membranes for use as peripheral nerve regeneration conduits. The surface and structure of the membranes were characterized, and nerve cell properties on the membrane surfaces were examined.

## 2. Results

### 2.1. Membrane Properties

**Figure 1:**
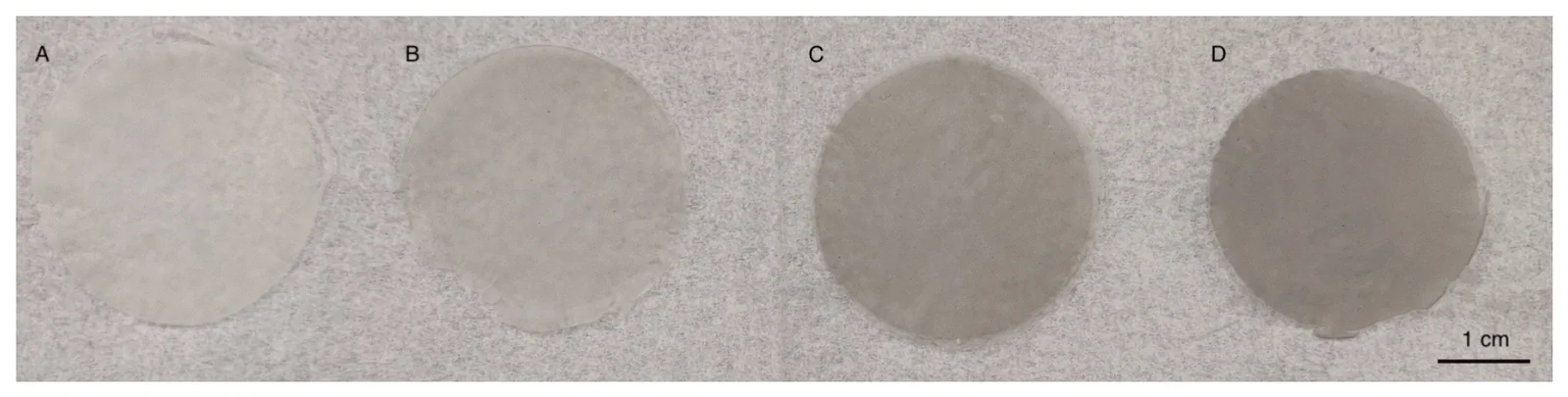
Digital images of the membranes prepared from chitosan (Ch), carboxymethylcellulose (CMC) and reduced graphene oxide (RGO). (**A**) ChCMC, (**B**) ChCMCRGO3, (**C**) ChCMCRGO6, (**D**) ChCMCRGO10.

**Figure 2:**
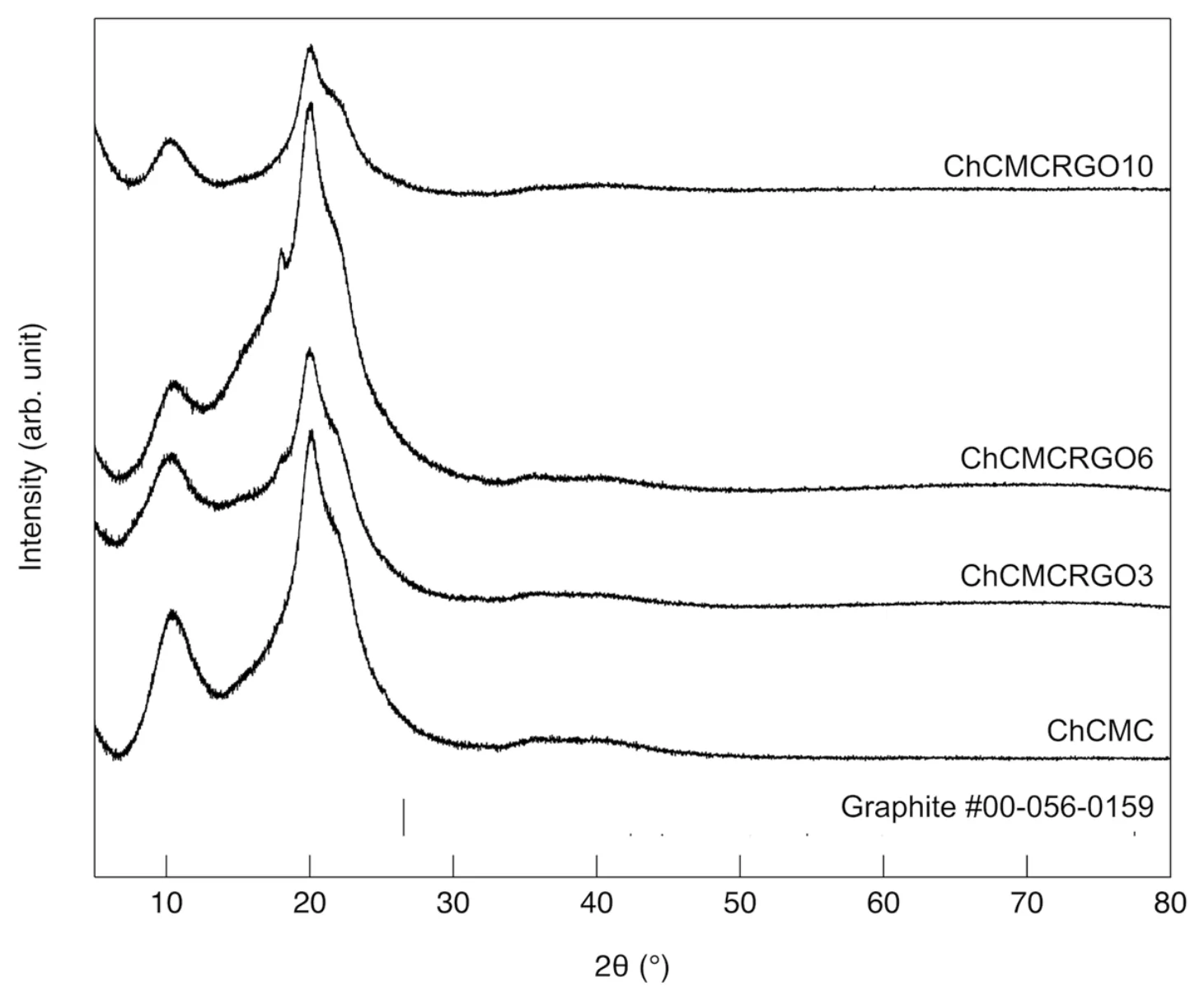
XRD patterns of ChCMCRGO membranes.

**Figure 3:**
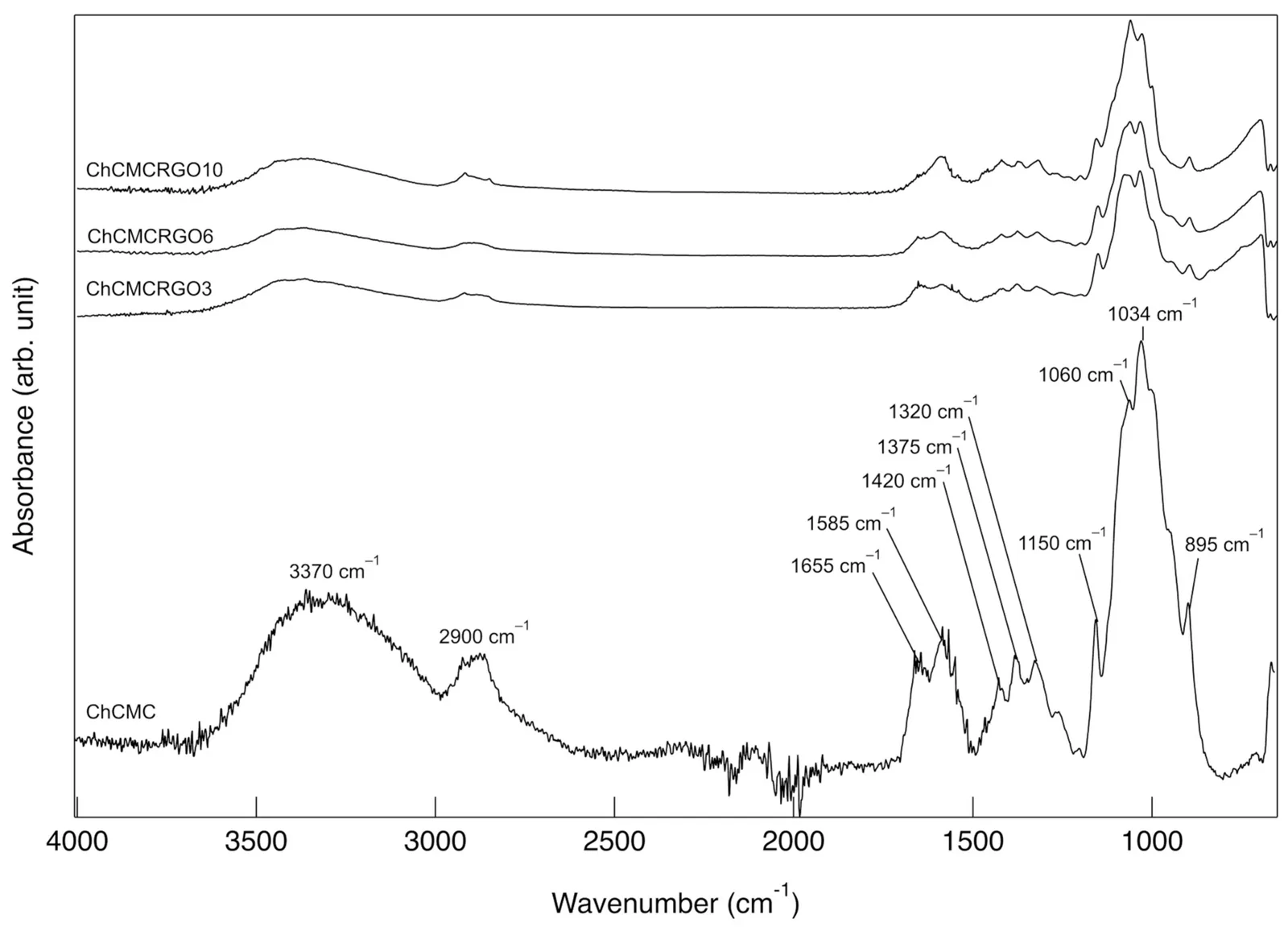
ATR-FTIR spectra of ChCMCRGO membranes.

Membranes were prepared from mixtures of Ch nanofibers, CMC nanofibers, and RGO at various compositions (Table 1), with higher concentrations of RGO yielding membranes increasingly gray in color (Figure 1). All membranes were 30–40 μm thick, had smooth surfaces, and were visually homogeneous. X-ray diffraction (XRD) patterns of ChCMC (Figure 2) showed that all the peaks corresponded to Ch (JCPDS #00-039-1894); 2θ = 10° (crystal plane 020), 15.2° (crystal plane 110), 20° (crystal plane 200), and 22.4° (crystal plane 220) [20]. Peaks corresponding to CMC and RGO were not detected. Figure 3 shows the attenuated total reflectance Fourier transform infrared (ATR-FTIR) spectra of the membranes. Ch was identified from its amine-related features at 1655 cm^−1^ (N–H deformation; amide II), 1587 cm^−1^ (N–H bending; amide I), and 1322 cm^−1^ (C–N stretching) [21,22]. CMC was characterized by carboxyl-related features at 1592 cm^−1^ (asymmetric stretching) and 1418 cm^−1^ (symmetric stretching) [23]. In the spectrum of ChCMC, the Ch-associated band observed at 1655 cm^−1^ shifted to 1645 cm^−1^, corresponding to a shift of approximately 10 cm^−1^. This shift is consistent with a change in the local environment of the Ch amino groups upon mixing with CMC. No additional peaks appeared upon the addition of RGO. Field emission scanning electron microscopy (FE-SEM) images of the membranes revealed a dense, fiber-like structure. Discrete RGO particles could not be clearly distinguished from the Ch/CMC nanofiber matrix under the present FE-SEM observation conditions. Therefore, the internal distribution, degree of dispersion, and connectivity of RGO within the membranes could not be determined from these images alone.

**Figure 4:**
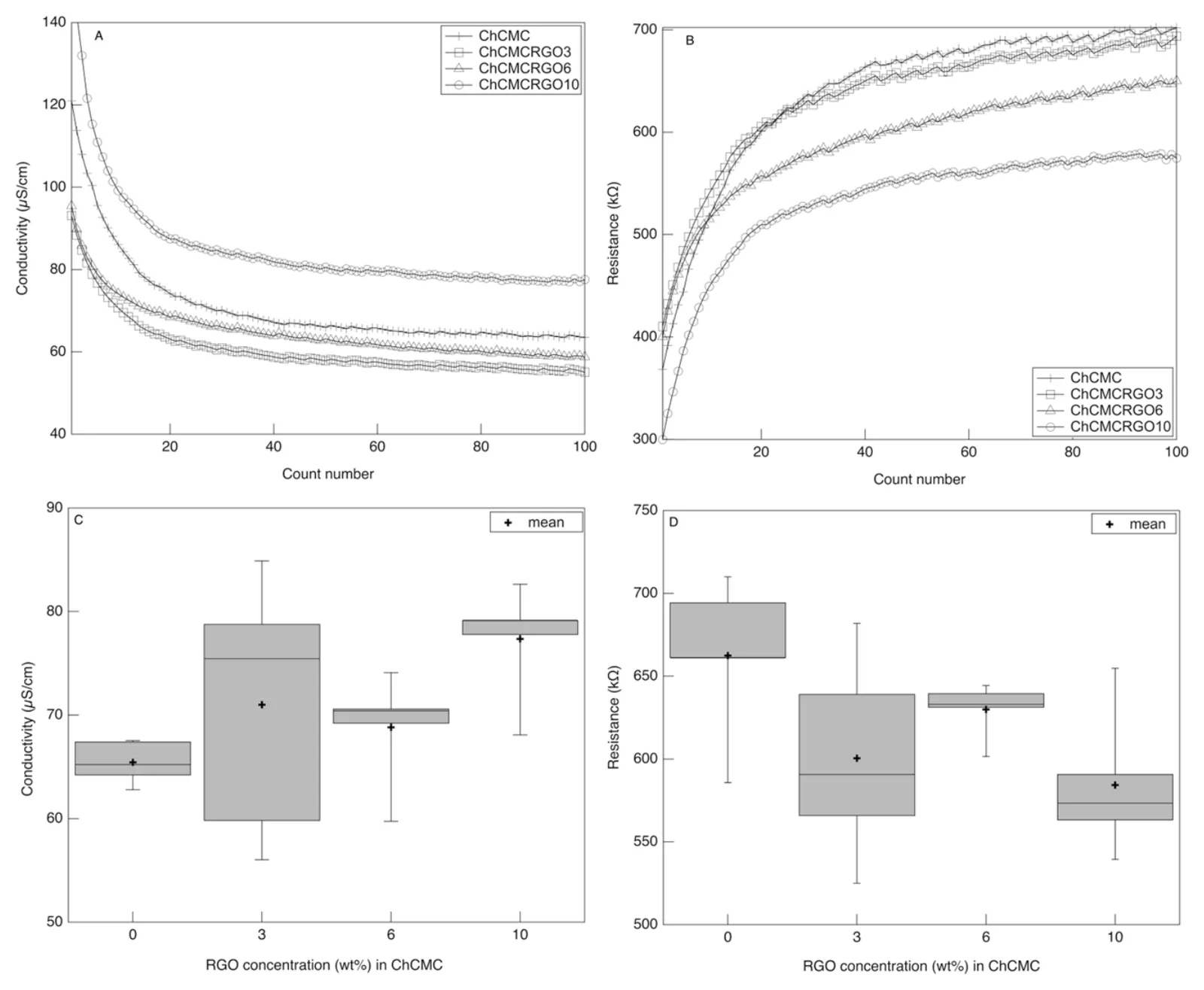
Time-dependent (**A**) conductivity and (**B**) resistance of membranes prepared with ChCMC and different concentrations of RGO and (**C**) average conductivity and (**D**) average resistance measured between 70 and 100 counts.

**Figure 5:**
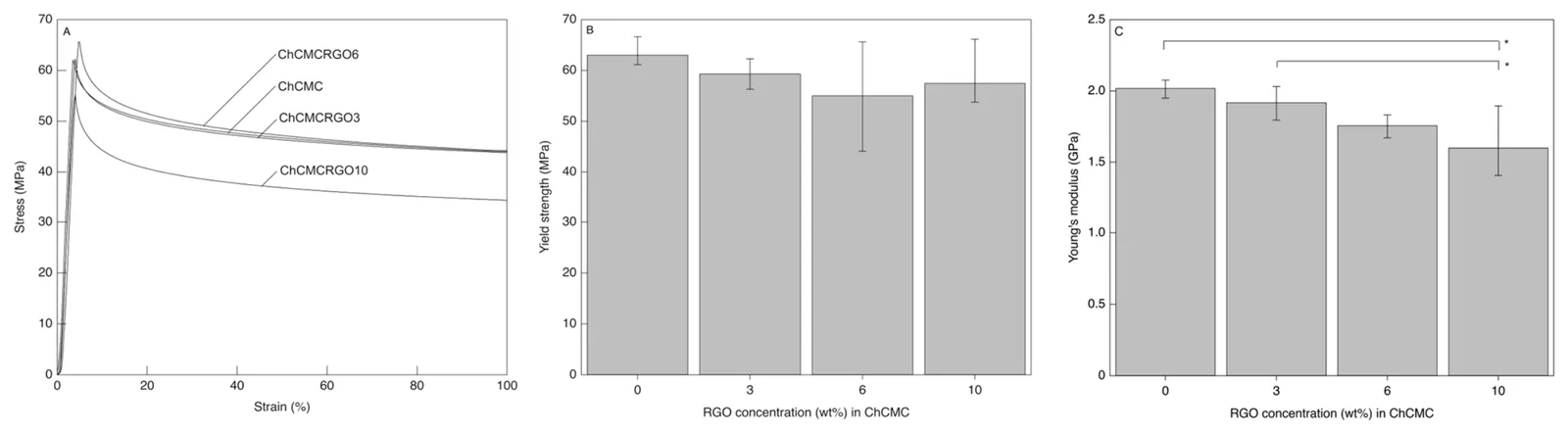
Mechanical properties of membranes prepared from ChCMC and different concentrations of RGO. (**A**) Stress–strain curve. (**B**) Yield strength. (**C**) Young’s modulus. * *p* < 0.05.

The water contact angle decreased monotonically as a function of RGO content from 95.3 ± 5.8°for ChCMC to 85.4 ± 7.5° for ChCMCRGO3, 80.5 ± 17.5° for ChCMCRGO6, and 65.3 ± 14.9° for ChCMCRGO10. The conductivity (Figure 4A) and resistance (Figure 4B) of the membranes initially varied markedly before becoming stable between 70 and 100 counts. Addition of RGO slightly increased the membrane conductivity from 65 μS/cm for ChCMC to 77 μS/cm for ChCMCRGO10 (Figure 4C) and decreased the resistance from 660 kΩ for ChCMC to 580 kΩ for ChCMCRGO10 (Figure 4D). However, these differences were not statistically significant among the tested membrane compositions. No abrupt increase in conductivity was observed with increasing RGO content, indicating that the present data do not provide evidence for the formation of an electrical percolation network within the investigated range of up to 10 wt% RGO. Regardless of the RGO content, all membranes had similar mechanical properties characterized by an initial linear elastic response to uniaxial stress followed by strain-softening behavior (Figure 5A). Greater RGO content resulted in a lower yield strength (Figure 5B) and a monotonic decrease in the Young’s modulus (Figure 5C), with ChCMC having a yield strength of 63 MPa and a Young’s modulus of 2 GPa and ChCMCRGO10 having a yield strength of 57.5 MPa and a Young’s modulus of 1.6 GPa. The mechanical properties reported herein refer exclusively to membranes tested in the dry state.

### 2.2. RT4-D6P2T Cell Morphology, Cytoskeletal Organization, and Proliferation on Membranes

The morphology, adhesion, and proliferation of RT4-D6P2T cells cultured on ChCMC membranes containing different amounts of reduced graphene oxide (RGO) were evaluated over 1, 3, 5, and 7 days in vitro (DIV).

**Figure 6:**
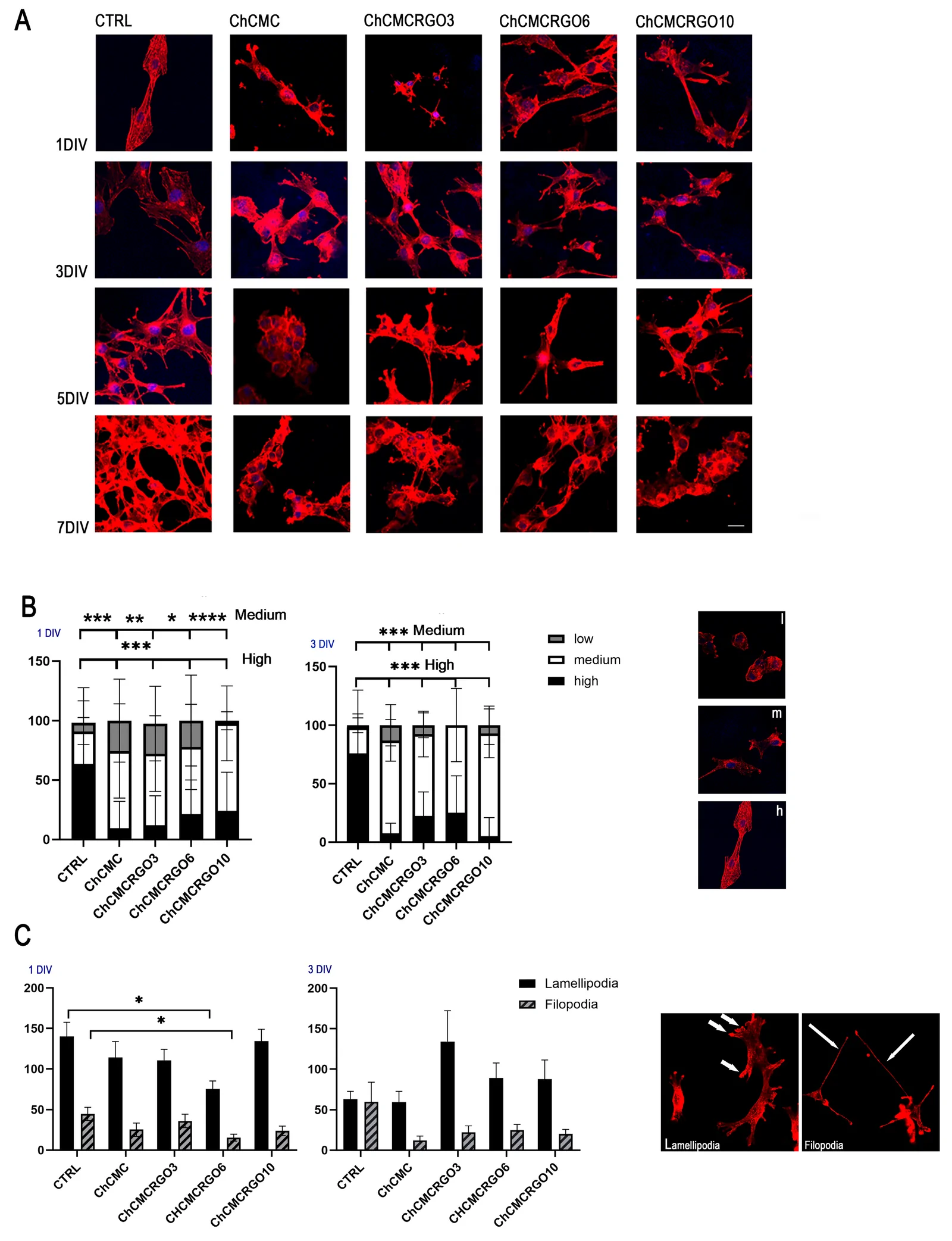
(**A**) Fluorescence images of RT4-D6P2T cells cultured on ChCMCRGO composite membranes for 1, 3, 5, and 7 days and stained with phalloidin (red) and DAPI (blue). Scale bar: 20 µm. (**B**) Quantification of actin cytoskeleton organization in RT4-D6P2T cells after 1 and 3 days of culture. Representative images of low (l), medium (m), and high (h) levels of actin cytoskeleton organization are shown. (**C**) Quantification of lamellipodia and filopodia protrusions in RT4-D6P2T cells after 1 and 3 days of culture. Arrows in the representative images indicate lamellipodia and filopodia. All statistical comparisons were performed against the control group. * *p* ≤ 0.05, ** *p* ≤ 0.01, *** *p* ≤ 0.001, and **** *p* ≤ 0.0001.

Representative fluorescence images of cells stained with phalloidin and DAPI are shown in Figure 6A. Cells adhered to all the tested membranes and progressively increased in number throughout the culture period, supporting the cytocompatibility of the materials. Changes in cell morphology were also observed in relation to membrane composition and culture time. In particular, cells cultured on membranes with higher RGO contents generally displayed a more spread morphology and a more evident organization of the actin cytoskeleton. At later time points, especially at 5 and 7 DIV, cells increasingly formed clusters, likely as a consequence of progressive proliferation and higher cell density.

To characterize early changes in cell spreading and cytoskeletal organization before extensive cell confluence occurred, actin organization was quantitatively evaluated at 1 and 3 DIV. Representative examples of cells classified as having low, medium, or high actin cytoskeleton organization and the distribution of the three morphological classes are shown in Figure 6B.

Cells cultured on glass coverslips exhibited a highly organized actin cytoskeleton, with clearly distinguishable stress fibers, cortical actin, lamellipodia, and filopodia. Compared with the control, cells cultured on the composite membranes showed a lower degree of cytoskeletal organization. At these time points, most cells on the membranes were classified as having an intermediate level of actin organization. Nevertheless, cells displaying poorly organized actin structures represented only a small fraction of the population, particularly visible on the ChCMCRGO10 membrane at 1 DIV and on the ChCMCRGO6 at 3 DIV, where no more than 2% of cells were assigned to the low-organization class.

**Figure 7:**
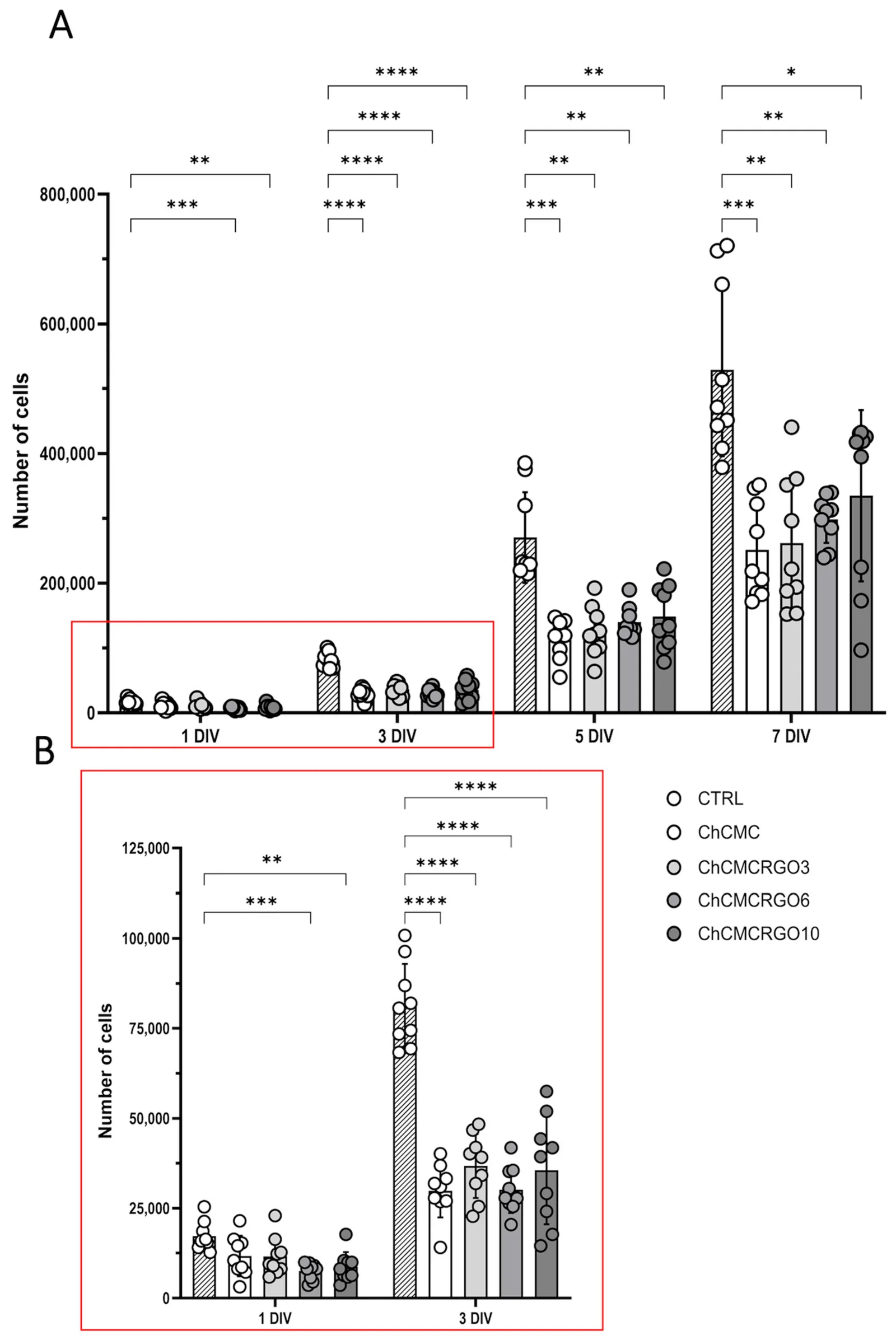
(**A**) Proliferation of RT4-D6P2T cells on control and on ChCMC composite membranes with RGO incorporation at different concentrations (3, 6, 10) after 1, 3, 5 and 7 days of seeding. (**B**) Bar graph providing an enlarged view of proliferation at 1 and 3 DIV. * *p* ≤ 0.05, ** *p* ≤ 0.01, *** *p* ≤ 0.001, **** *p* ≤ 0.0001.

Representative images highlighting lamellipodia and filopodia are shown in Figure 6C. Quantification of these actin-based protrusions revealed comparable numbers of lamellipodia and filopodia in the control and membrane-cultured cells at both 1 and 3 DIV (Figure 6C), with the exception of the ChCMCRGO6 group at 1 DIV, which showed a significant reduction compared with the control in terms of lamellipodia and filopodia formation. Cell proliferation was assessed by determining the total number of cells at 1, 3, 5, and 7 DIV (Figure 7). Cells cultured on glass coverslips showed the highest proliferation rate throughout the experimental period. However, a progressive and sustained increase in cell number was also observed on all the composite membranes. Notably, the differences in cell number between the control and the membrane groups became less pronounced after 7 days of culture than at the earlier time points.

### 2.3. Protein Expression in RT4-D6P2T Cells Cultured on Membranes

**Figure 8:**
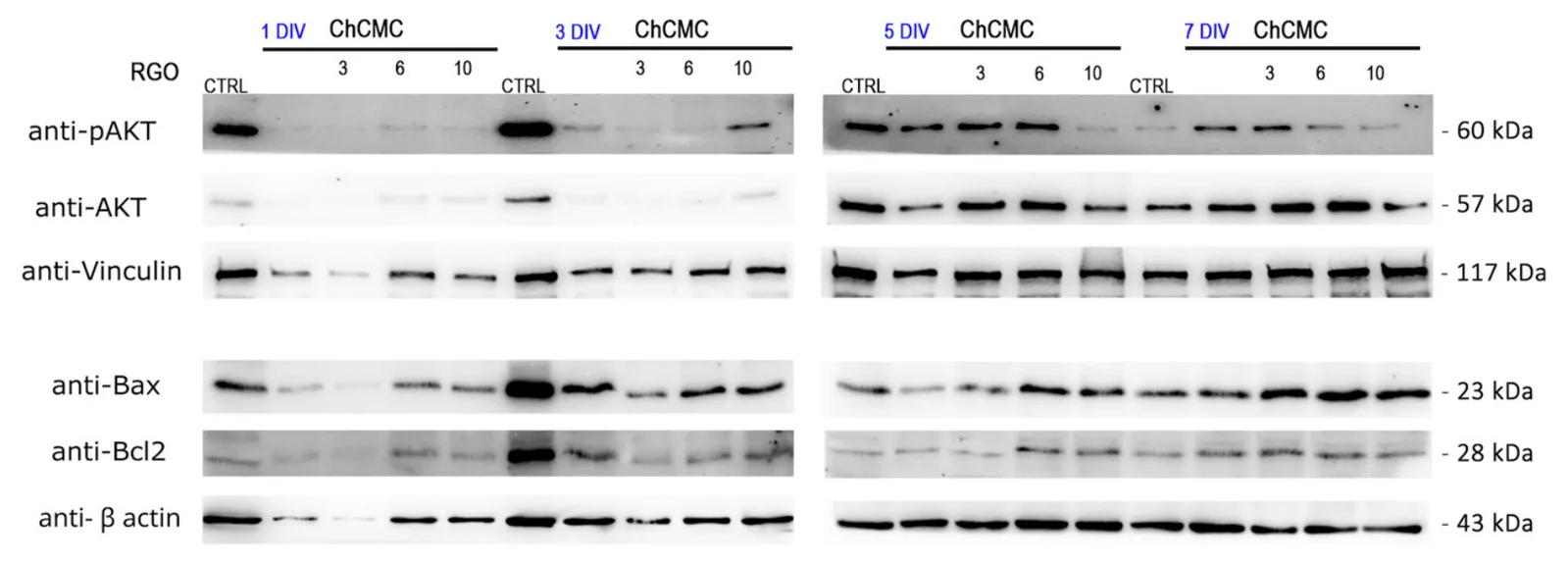
Western blot analysis of the expression of pAKT, AKT, bcl-2, and bax proteins on RT4-D62T cells cultured for 1, 3, 5, and 7 days in vitro (DIV) on ChCMCRGO3/6/10 composite membranes. Vinculin was used as a loading control for high molecular weight proteins and β-actin for low molecular weight proteins. Protein extracts were pooled for each condition and time point, thus providing a qualitative overview that does not reflect inter-sample variability (Supplementary Materials).

The molecular response of RT4-D6P2T cells cultured on the composite membranes was explored by Western blotting. Protein extracts from multiple samples were pooled for each experimental condition and collected after 1, 3, 5, and 7 DIV; therefore, the blots shown in Figure 8 provide a qualitative overview and do not reflect inter-sample variability.

Total AKT remained detectable throughout the experimental period, although variability in band intensity was observed among conditions and time points. The pAKT signal appeared weaker in cells cultured on the ChCMCRGO6 and ChCMCRGO10 membranes at 5 and 7 DIV. However, because reliable densitometric quantification could not be performed, these observations should be considered exploratory and do not support quantitative conclusions regarding total AKT expression or AKT activation. Overall, the continued detection of the examined proteins provides qualitative molecular context that is broadly consistent with the maintenance of cell viability and function on the biomaterials observed in the other biological assays, including an initial adaptation phase followed by improved cellular responses. To further explore the cellular response to culture on the composite membranes, the pro-apoptotic protein Bax and the anti-apoptotic protein Bcl-2 were also examined. Both proteins remained detectable across the investigated membrane compositions and culture times, and visual inspection did not reveal large or consistent differences in their band-intensity patterns. These qualitative observations are broadly consistent with the results of the other biological assays, which indicated the maintenance of cell viability on the biomaterials following an initial adaptation phase. Nevertheless, in the absence of reliable densitometric quantification, moderate changes in Bax and Bcl-2 expression or in their relative balance cannot be excluded.

### 2.4. DRG Neurite Outgrowth on Membranes

**Figure 9:**
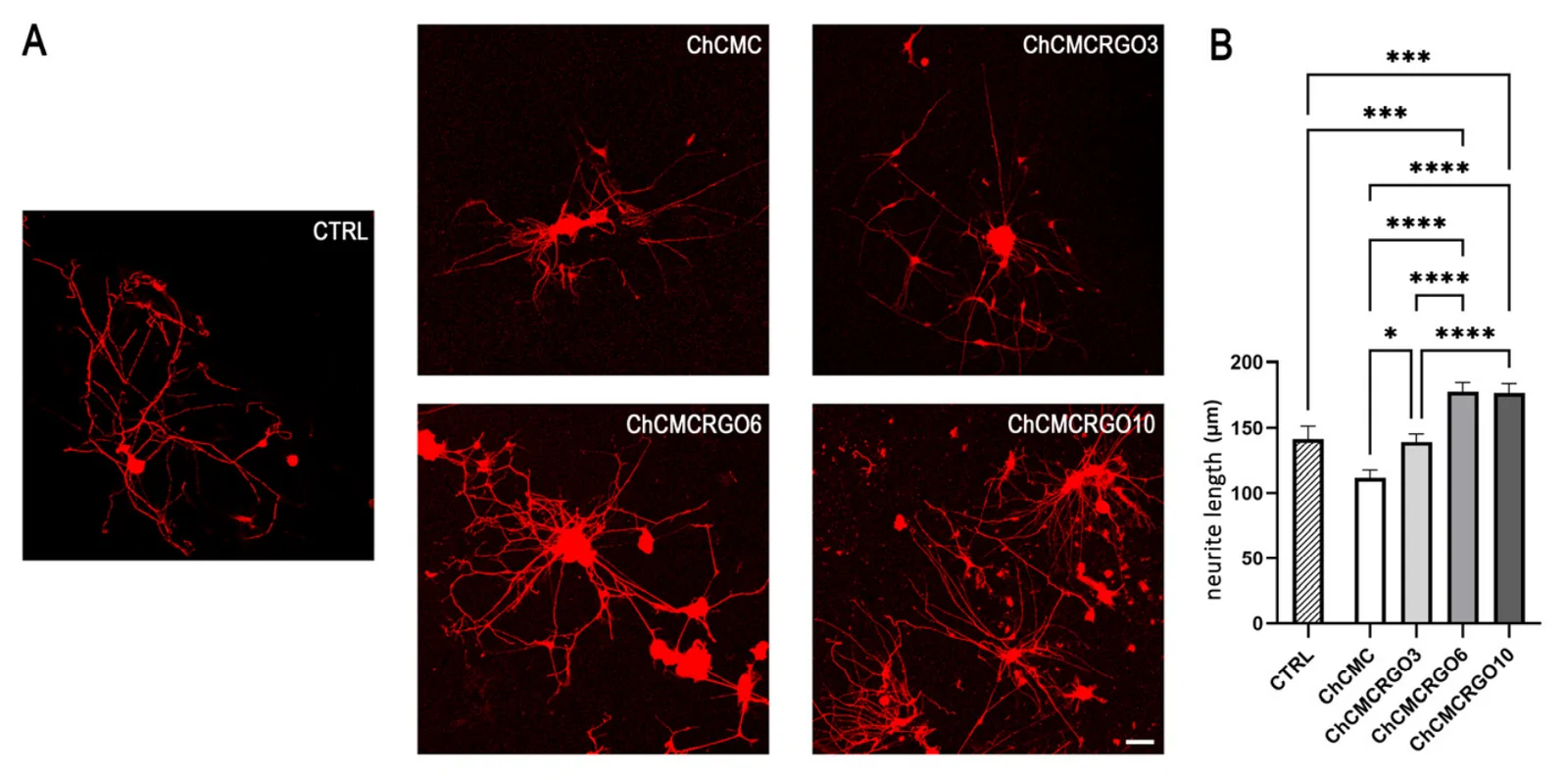
(**A**) Representative images of DRG sensory neurons cultured on control and on ChCMCRGO composite membranes for 3 days, stained with β-tubulin III (red). Scale bar: 50 µm. (**B**) Bar graph representing the evaluation of the neurite extension of the DRG neurons cultivated on ChCMCRGO composite membranes. * *p* ≤ 0.05, *** *p* ≤ 0.001, **** *p* ≤ 0.0001.

In Figure 9, it is possible to observe fluorescence images of dissociated DRG neurons cultured on glass slides or on the different composite membranes, stained with the β-tubulin marker (Figure 9A). The biocompatibility was excellent, so we were able to analyze the extent of neurite length. Neurite length increased with increasing RGO content.

The graph shows that neurons cultured on RGO-containing membranes exhibit a significant increase in neurite outgrowth compared with those in direct contact with membranes without RGO. Interestingly, the membranes with the highest RGO content, ChCMCRGO6 and ChCMCRGO10, also show a significant increase in neurite length compared with the control samples (Figure 9B).

## 3. Discussion

Filtration proved to be the best method for obtaining visually homogeneous, strong, and intact ChCMC nanofiber membranes that can readily be rolled to create a conduit for peripheral nerve regeneration. Ch and CMC nanofibers in the composite films were well mixed, and no phase separation occurred. Addition of RGO did not change the aforementioned properties, with the membranes remaining visually homogeneous, strong, and intact, although the color gradually turned grayer at higher RGO concentrations. The electrical conductivity of the composite membranes was improved by the inclusion of up to 10 wt% RGO. Although a higher RGO content could further increase the electrical conductivity, the membranes became more brittle and fragile at RGO contents above 10 wt%.

Surface characterization using ATR-FTIR did not identify any differences among the spectra of the composite membranes with different RGO concentrations, indicating that no chemical bonding occurred in the presence of RGO. The carboxyl groups of CMC were found to exist as –COO^−^ coupled to Na^+^. The amino groups of chitosan nanofibers were present mostly as –NH_2_, with a small protonation of –NH_3_^+^ at a neutral pH. The Ch-associated band observed at 1655 cm^−1^ shifted to 1645 cm^−1^ after the formation of the ChCMC membrane. This approximately 10 cm^−1^ shift indicates a change in the chemical environment of the Ch amino groups and is consistent with electrostatic interactions between protonated amino groups of Ch and carboxylate groups of CMC [20]. However, because no additional spectroscopic analysis was performed to resolve all individual band shifts in the composite, the FTIR results should be interpreted as supporting, rather than definitively proving, electrostatic complexation between Ch and CMC. ATR-FTIR analysis also showed that the incorporation of RGO into the composite membranes resulted in no additional peaks, indicating that no new chemical bonds were formed. The broad XRD peaks characterizing individual crystal phases indicated that the Ch and CMC membranes were semi-crystalline. After mixing Ch and CMC, the crystallinity of CMC was greatly reduced, as evidenced by the absence of the characteristic peak at 2θ = 12°. Instead, the only visible peaks corresponded to the Ch crystalline phase (2θ = 10°, 15.2°, 20° and 22.4°), indicating that amorphous CMC nanofibers were dispersed among semi-crystalline Ch nanofibers. The intensities of peaks at 2θ = 10° and 2θ = 20° were lower for membranes that contained RGO than those that did not, but no new peaks appeared in the presence of RGO, which always exists as an amorphous material with weak diffraction peaks compared with graphite [24]. This suggests that the Ch nanofibers were arranged in a semi-crystalline structure surrounded by amorphous CMC nanofibers. The absence of an RGO-dependent shift in the Ch diffraction peaks suggests that incorporation of RGO did not markedly alter the crystalline structure of the Ch nanofibers. However, XRD alone does not provide sufficient information to determine the spatial distribution or interfacial arrangement of RGO within the Ch/CMC matrix. The surface wettability of the membranes was investigated by measuring the water contact angle. RGO has some affinity for water owing to the presence of hydroxyl groups on the graphene sheets. Therefore, the wettability of composite membranes monotonically decreased as a function of RGO content. According to Lee et al., cells grown on a moderately hydrophilic surface (contact angle between 60° and 70°) have greater proliferation and neurite formation than those grown on extremely hydrophilic (contact angle under 50°) or hydrophobic (contact angle above 90°) surfaces [25]. Among the tested composites, ChCMCRGO10 had the most suitable water contact angle, with an average value of approximately 70°.

Ch, CMC and ChCMC membranes yielded stress–strain curves typical of plastic materials. ChCMC composite membranes had a higher yield strength and Young’s modulus than Ch or CMC membranes, which can be explained by the electrostatic interactions between –NH_3_^+^ and –COO^−^ groups on Ch and CMC, respectively. RGO incorporation did not markedly alter the overall shape of the stress–strain curves. Yield strength showed no significant change among the tested compositions, whereas Young’s modulus decreased with increasing RGO content. These results suggest that RGO incorporation primarily affected membrane stiffness rather than strength, which should be considered in the development of nerve-guidance materials requiring an appropriate balance of mechanical integrity and flexibility. A limitation of the present study is that mechanical testing was performed only in the dry state. Therefore, wet state mechanical properties, suturability, and cyclic deformation performance under physiologically relevant conditions remain to be investigated in future studies. A slight increase in conductivity was observed with increasing RGO content. However, no abrupt change characteristic of a clear electrical percolation threshold was detected across the investigated compositions. This behavior may reflect partial electrical connectivity between RGO domains within the Ch/CMC nanofiber matrix. However, because the internal distribution and connectivity of RGO could not be directly determined from the present structural analyses, the relationship between RGO organization and electrical conductivity cannot be established from the present data.

Schwann cells are essential for nerve regeneration, as they produce the myelin sheath that protects the axon [26]. RT4-D6P2T is a Schwann cell line generally obtained from rat neurotumors and is commonly used to study the behavior of peripheral nerve system cells.

The present study evaluated the biological response to ChCMCRGO composite membranes containing increasing amounts of reduced graphene oxide. Overall, all formulations supported RT4-D6P2T cell adhesion, cytoskeletal organization, and proliferation, indicating good cytocompatibility.

Although cells cultured on glass exhibited a more organized actin cytoskeleton and a higher proliferation rate, the number of cells on the composite membranes progressively increased over time. The lower initial cell number observed on the membranes may be related to differences between the glass and ChCMCRGO surfaces in terms of stiffness, roughness, wettability, protein adsorption, or initial cell-seeding efficiency. These material properties are known to influence cell adhesion and proliferation and may therefore contribute to the different kinetics observed during the early culture period [27,28]. Moreover, the initial delay in cell adhesion and proliferation may reflect the time required for cells to adapt to the new substrate [29].

Most membrane-cultured cells displayed an intermediate level of actin organization, while poorly organized cells represented only a small fraction. Lamellipodia are protrusions at the leading edge of a cell, primarily composed of a dense network of actin filaments. These structures are involved in cell migration and play a crucial role in tissue development [30]. They enable the cell to spread and move by extending and attaching to the substrate. Filopodia are slender protrusions extending from the surface of a cell, consisting of parallel bundles of actin filaments. These structures are critical for sensing the cellular environment, guiding cell migration, and establishing connections with other cells or the extracellular matrix. Filopodia help cells explore their surroundings and make directional decisions during movement [31]. Lamellipodia and filopodia formation was generally comparable to the control, suggesting that the membranes did not markedly impair cell spreading or cell–substrate interactions. The temporary reduction observed on ChCMCRGO6 at 1 DIV may reflect an early adaptive response to the material surface.

Cells cultured on membranes with higher RGO content generally appeared more spread and showed a more evident actin organization. This effect may be related to RGO-induced changes in surface topography, mechanical properties, protein adsorption, or electrical characteristics, although further studies are required to identify the mechanisms involved.

Western blot analysis provided only qualitative information and did not reveal marked differences in Bax and Bcl-2 band patterns among formulations or culture times. This observation was consistent with the viability and proliferation results but could not exclude moderate changes in apoptotic signaling. Further analyses using optimized protein extraction and quantification protocols are required. The most relevant finding was the enhancement of DRG neurite outgrowth on RGO-containing membranes. Neurite length increased with RGO content, and ChCMCRGO6 and ChCMCRGO10 promoted greater neurite extension than both RGO-free membranes and glass controls. This effect may be associated with physicochemical changes introduced by RGO, including changes in surface wettability, topography, protein adsorption, and electrical properties [19]. However, because the conductivity increase was modest and no external electrical stimulation was applied in the present study, the enhanced neurite outgrowth cannot be attributed specifically to electrical conductivity. Further studies combining controlled electrical stimulation with materials of systematically varied conductivity will be required to determine whether electrical properties directly contribute to the observed neuronal response.

Overall, the results show that ChCMCRGO membranes are cytocompatible and support neuronal growth. In particular, the higher RGO formulations appear promising for peripheral nerve tissue-engineering applications. Future studies will focus on further optimizing the device to enhance its electrical conductivity, potentially through the functionalization of the membrane surface with RGO. In parallel, an aligned surface topography will be developed to promote directional cell migration and guide axonal regrowth. These improvements will contribute to refining the device as a multifunctional platform for peripheral nerve repair.

## 4. Materials and Methods

### 4.1. Materials

Solutions of Ch nanofibers (2 wt%) and CMC nanofibers (2 wt%) were purchased from Sugino Machine Limited (Toyama, Japan). Reduced graphene oxide (RGO) suspension was supplied by Nippon Shokubai Co., Ltd. (Tokyo, Japan).

### 4.2. Membrane Preparation

Ch and CMC nanofiber solutions were mixed in a 7:3 weight ratio, and then RGO was added to the nanofiber solution at 0, 3, 6, and 10 wt% relative to the Ch and CMC, as shown in Table 1. The mixture was filtered with a cellulose nitrate membrane (pore diameter, 0.2 μm; Cytiva, Little Chalfont, UK) using a vacuum pump (LV-125, Nitto Kohki Co., Ltd., Tokyo, Japan). After filtration, the membranes were dried at 40 °C in an oven (Constant Temperature Oven, Yamato Scientific, Tokyo, Japan) for 2 h and then the filter was removed. The compositions and sample name are described in Table 1.

### 4.3. Surface Characterization

The crystal phase of the membranes was studied using a thin-film X-ray diffractometer (Rigaku Smart Lab, Tokyo, Japan) with a CuKα X-ray source, 45 kV, 200 mA, continuous scanning, and an angular increment of 0.01°. The chemical structure of the membranes was examined using an ATR-FTIR spectroscopy (IR Tracer-100, Shimadzu, Kyoto, Japan) with a resolution of 4 cm^−1^ and 64 accumulated scans. The wettability of the membranes was evaluated at room temperature using the sessile drop method with a contact angle goniometer (100SB, Sindatek, Taipei City, Taiwan) and 10 drops of distilled water. Surface morphologies were observed using FE-SEM (JSM-6701F0N0, JEOL, Tokyo, Japan) at an accelerating voltage of 5 kV after sputtering a 12.5 nm Pt-Pd layer (12.5 nm) on each membrane.

### 4.4. Mechanical Properties

The membranes were cut into thin rectangular (1 cm × 3 cm) pieces (*n* = 4). To attach the pieces to the clamps of a creep-meter (RHEONER II, Yamaden, Tokyo, Japan), paper tabs (1 cm × 2 cm) were adhered to both ends of each membrane, leaving a central surface area of 1 cm × 1 cm for analysis. The distance between the clamps was 10 mm and the measuring speed was 0.5 mm/s. The mechanical strength was evaluated using the yield strength and Young’s modulus. The Young’s modulus was determined from the slope of the initial linear region of the stress–strain curve. The value reported as yield strength was taken as the maximum stress observed in the stress–strain curve.

### 4.5. Electrical Conductivity

The electrical conductivity of the membranes was measured using a four-point probe (LRS4-T, Keithlink, Taipei City, Taiwan). Samples measuring 1 cm × 1 cm (n = 5) were placed under the probe. Membrane thickness was measured using calipers. An electrical current was run through the sample and the recorded tension and intensity were used to calculate the sheet resistance, volume resistivity, resistance and conductivity, as detailed in Equations (1)–(4). Each measurement was made for 100 counts, and data acquired between 70 and 100 counts was used for the calculations.

(1) $$Sheet\;resistance\;(\Omega /sq)=\frac{tension\;(V)\times \pi }{current\;(A)\times ln2}$$

(2) $$Volume\;resistivity\;\left(\Omega \times cm\right)=sheet\;resistance\times sample\;thickness\;(cm)$$

(3) $$Resistance\;\left(\Omega \right)=Volume\;resistivity\times \frac{sample\;length\;(cm)}{sample\;width\;\left(cm\right)\times sample\;thickness\;(cm)}$$

(4) $$Conductivity\;\left(S/cm\right)=\frac{1}{volume\;resistivity}$$

### 4.6. In Vitro RT4-D6P2T Analyses

Membrane samples were cut into circles with a diameter of 1.2 cm and sterilized with ethylene oxide gas (Kapox 20% concentration, temperature 45 °C, humidity 50%, 3 h, Steritek Corporation, Saitama, Japan). RT4-D6P2T cells (ATCC-catalog no. CRL-2768) were seeded at a density of 5000 cells/cm^2^ on the samples and on control wells and cultivated in Dulbecco’s Modified Eagle’s Medium (DMEM, Sigma-Aldrich, St. Louis, MO, USA), containing 4500 mg/L glucose, L-glutamine and sodium bicarbonate, supplemented with 10% heat-inactivated fetal bovine serum (FBS, Gibco™, Thermo Fisher Scientific, Waltham, MA, USA), 1% penicillin-streptomicin (P/S, PAN Biotech™, PAN-Biotech GmbH, Aidenbach, Germany), 1% glutamine (Q,) and 1% sodium-pyruvate (NaPyr, Gibco™). For the proliferation analysis, after 1, 3, 5, and 7 days in vitro (DIV), the cells were detached from the films by soaking in 80 µL of trypsin (Trypsin-EDTA solution 1×, Sigma™) for 5 min (37 °C, 5% CO_2_) and suspended in 330 µL of DMEM and counted in a Burker chamber. The RT4-D6P2T proliferation experiment was performed on ChCMC, ChCMCRGO3, ChCMCRGO6, ChCMCRGO10, and on an uncoated glass coverslip. This experiment was performed in technical and biological triplicate. The counts obtained were analyzed, mean-valued, and expressed as the total number of cells ± standard deviation.

For the morphological evaluation of the actin cytoskeleton, cells were cultured on the samples for 1, 3, 5, and 7 days and incubated at 37 °C, 5% CO_2_ atmosphere at an initial density of 3000 cells/cm^2^.

At the referred timepoints, cells were washed with PBS (Sigma-Aldrich) and fixed with a 4% paraformaldehyde solution, then permeabilized for 1 h at room temperature using PBS 0.1% Triton. Phalloidin (Thermo Fisher Scientific™), diluted at 1:400 in PBS, and DAPI, diluted at 1:1000 in PBS, were used to identify the actin cytoskeleton and nuclei, respectively. This was achieved by incubating the sample for 1 h at room temperature, without any light, followed by three washes with PBS, and mounting the samples using Dako fluorescent mounting medium (Sigma-Aldrich).

On Day 1, each sample was photographed in thirty different fields of view, while at Days 3, 5 and 7, photographs of samples were acquired in ten different fields. Images were captured using a Zeiss LSM800 confocal laser microscope (Carl Zeiss Microscopy GmbH, Jena, Germany). The number of cells in each image was estimated using the Fiji (ImageJ distribution, version 20250529-2217; National Institute of Health, Bethesda, MD, USA).

In order to accomplish a quantitative analysis of cell morphology, the organization of the actin cytoskeleton was assessed. To each cell was assigned an arbitrary score based on its actin cytoskeleton organization. These scores were categorized into three levels: poor, medium, or high actin cytoskeleton organization (Figure 6B). The number of cells in each category (poor, medium, high) was calculated and expressed as a percentage.

Additionally, the development of lamellipodia and filopodia was evaluated. Lamellipodia are broad, sheet-like cellular protrusions, while filopodia are slender, rod-like protrusions (Figure 6C). The percentage of cells in each category was determined to express the results quantitatively. This structured approach ensures a comprehensive quantitative analysis of cell morphology based on actin cytoskeleton organization and the development of cellular protrusions, contributing to the understanding of cellular behaviors and properties in the given experimental context.

### 4.7. Biomolecular Analysis

#### 4.7.1. Protein Extraction

For protein extraction from RT4-D6P2T cells cultured on the different types of membranes and on plastic as controls, cells were cultured in a 24-well plate for 1 and 3 days at a density of 30,000 cells/cm^2^ and for 5 and 7 days at a density of 20,000 cells/cm^2^. Cells were detached with 200 µL of trypsin (Sigma-Aldrich) for 5 min (37 °C, 5% CO_2_), then inactivated with 700 µL of 10% FBS- containing DMEM. Subsequently, cells were collected in a pool for each condition and centrifuged at 1200 rpm for 4 min at RT to obtain a pellet. The supernatant was removed, and total proteins were extracted by suspending cells in 200 µL of boiling Laemmli buffer (2.5% SDS, 0.125 M trisHCl pH 6.8, Sigma-Aldrich) and boiling them for 3 min at 100 °C. The extracted material was passed through a 32 G needle several times to disrupt genomic DNA. The Eppendorf tubes containing protein extracts were centrifuged for 10 min at maximum speed at RT to collect the supernatant, discarding cell debris. The samples were stored at −20 °C.

Protein quantification was performed using the Bicinchoninic Acid assay kit (Sigma), and equal amounts of protein (30 µg) were loaded separated by 4–20% SDS-PAGE.

#### 4.7.2. Western Blot

Proteins were extracted from RT4-D6P2T cells cultured on different films by pooling four samples per condition. Protein expression was then analyzed by Western blotting. Samples were separated on 4–20% gradient precast gels (Mini-PROTEAN TGX Stain-Free, Bio-Rad Laboratories, Inc., Hercules, CA, USA) or 15% acrylamide gels for low-molecular-weight proteins. Proteins were transferred onto nitrocellulose membranes at 350 mA for approximately 1 h at 4 °C. Transfer efficiency was verified by Ponceau S staining (Sigma-Aldrich). Membranes were blocked with 5% non-fat dry milk in TBST for 1 h at room temperature and incubated overnight at 4 °C with primary antibodies. After washing, membranes were incubated for 1 h with HRP-conjugated anti-rabbit or anti-mouse secondary antibodies (Cell Signaling, Danvers, MA, USA, #7074 and #7076; 1:15,000). Chemiluminescent signals were developed using Clarity Western ECL or Clarity Max Western ECL substrates (Bio-Rad) and acquired with a ChemiDoc Touch Imaging System. When required, HRP was inactivated with 0.02% NaN_3_ in TBST before membrane reprobing. Primary and secondary antibodies are listed in Table 2.

### 4.8. DRG Harvesting, Neuron-Dissociation, Culture and Immunofluorescence

These experimental procedures complied with European Union Directive 2010/63/EU and were approved by the Ethics Committee of the University of Turin before the study began (Italian Ministry of Health, project no. 690/2025-PR). Female Adult Wistar rats (Envigo Laboratories, Milan, Italy) (n = 2, 250 g in weight) were used. Animals were housed in large same-sex cages at the animal facility of Neuroscience Institute Cavalieri Ottolenghi (NICO; Ministerial authorization DM 182 2010-A 3-11-2010) in humidity- and temperature-controlled room with 12-h light/dark cycles. Dorsal root ganglia (DRGs) were isolated from rats following lethal anesthesia with ketamine and xylazine. After vertebral column dissection, the spinal canal was opened and DRGs were identified along the dorsal roots and collected. DRGs were enzymatically dissociated in F12 medium with collagenase type IV (12.5 mg/mL) for 1 h (Sigma-Aldrich), followed by an additional 45 min incubation, and then treated with bovine pancreatic trypsin (2 × 25 mg/mL) for 30 min at 37 °C and 5% CO_2_. Trypsin was inactivated with 33% fetal bovine serum. Ganglia were mechanically dissociated by gentle trituration, filtered through a 70-μm cell strainer (BD Falcon™, Corning Inc., Corning, NY, USA), and centrifuged. DRG neurons were purified by differential centrifugation through 30% bovine serum albumin and resuspended in modified Bottenstein and Sato’s medium containing N2, AraC, and insulin (Sigma Aldrich).

Dissociated cells were cultured for 72 h on glass coverslips (CTRL) or laminin-coated films (2 μg/cm^2^) (ChCMC, ChCMCRGO3, ChCMCRGO6, ChCMCRGO10) at 37 °C and 5% CO_2_. Six technical replicates were performed for each biological replicate. Cultures were fixed with 4% paraformaldehyde, permeabilized with 0.1% Triton X-100, and immunostained with mouse anti-βIII-tubulin (1:1.000; Sigma-Aldrich) followed by a Cy3-conjugated secondary antibody (Jackson ImmunoResearch Laboratories, Inc., West Grove, PA, USA).

Images were acquired using a Zeiss LSM800 confocal microscope and analyzed with Fiji (ImageJ distribution). Cells bearing multiple neurites were classified as differentiated. Neurite length was measured only for processes at least twice the cell-body diameter.

### 4.9. Statistical Analysis

For statistical analysis, data were analyzed by GraphPad Prism, version 10.6.1 (GraphPad software LLC, Boston, MA, USA), using one-way ANOVA followed by Tukey tests. The level of significance was set at *p* ≤ 0.05 (*), *p* ≤ 0.01 (**), *p* ≤ 0.001 (***), and *p* ≤ 0.0001 (****). Data were expressed as mean ± standard deviation.

## Figures and Tables

**Table 1:** The sample name and the amount of reduced graphene oxide (RGO) in ChCMC nanofiber solution.

Sample Name	RGO (wt%)
ChCMC	0
ChCMCRGO3	3
ChCMCRGO6	6
ChCMCRGO10	10

**Table 2:** Protein targets and primary antibodies used for Western blot.

Protein Target	Primary Antibody, Species, Dilution, Catalog No, Manufacturer
pAKT	α-pAKT Mouse (1:1000), #40515, Cell Signaling Technology
AKT	α-AKT Rabbit (1:1000), #92725, Cell Signaling Technology
Bax	α-Bax Rabbit (1:2000), 50599_2, Cell Signaling
Bcl-2	α-Bcl2 Mouse (1:200), SC7382, Abcam (Cambridge, UK)
β-actin	α-βactin Mouse (1:1000), SC47778, Sigma
Vinculin	α-vinculin Mouse (1:2000), V9131-100U, Sigma-Aldrich

## Data Availability

The data presented in this study are available within the article.

## Supplementary Materials

The following supporting information can be downloaded at: https://www.mdpi.com/article/10.3390/ijms27188013/s1.
